# Supplementary material for: Green Synthesis and Electrochemical Properties of Mono- and Dimers Derived from Phenylaminoisoquinolinequinones
Source: Molecules. 2019 Nov 30;24(23):4378. doi: 10.3390/molecules24234378 (PMC6930604; doi:10.3390/molecules24234378)
Supplement: Supplementary file 1 [file molecules-24-04378-s001.pdf]

Green Synthesis and Electrochemical Properties of Mono- and Dimers Derived from Cytotoxic Phenylaminoisoquinolinequinones

Juana Andrea Ibacache [1]*, Jaime A. Valderrama [2]*, Judith Faúndes [1], Alex Danimann [1], Francisco J. Recio [3] and César Zúñiga [3]

[1] Facultad de Química y Biología, Universidad de Santiago de Chile, Alameda 3363, casilla 40, Santiago, 9170022, Chile; judith.faundes@usach.cl (J.F.); alex.danimann@usach.cl (A.D.).
[2] Facultad de Ciencias de la Salud, Universidad Arturo Prat, casilla 121, Iquique, Chile, 1100000
[3] Facultad de Química y Farmacia, Universidad Católica de Chile, casilla 306, Santiago, Chile, 7820436; czuniga1@uc.cl (C.Z.); javier.recio@uc.cl (F.R.).

* Correspondence: juana.ibacache.r@usach.cl (J.A.I.); jaimeadolfov@gmail.com (J.A.V.).

Key words: Twin drugs; heterodimers; green synthesis; amination reaction; cyclic voltammetry; half wave potential.

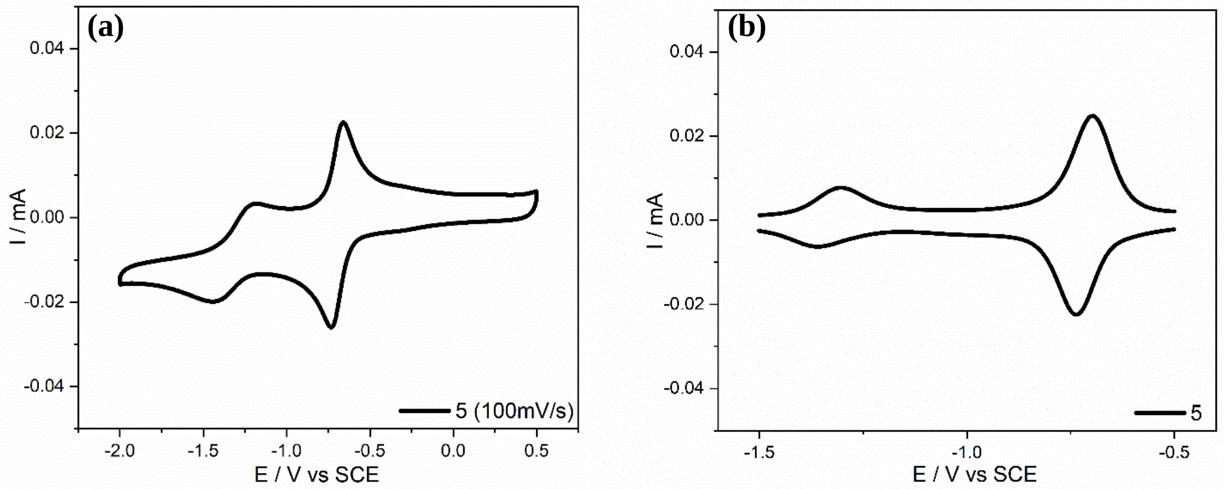

Figure S1. (a) Cyclic voltammetry 100mV s^{-1} and (b) square wave voltammetry (SWV) with a concentration of 0.1M of the monomer **5**, in 0.1 M tetrabutylammonium perchlorate (TBAP) in acetonitrile and saturated N_2 atmosphere.

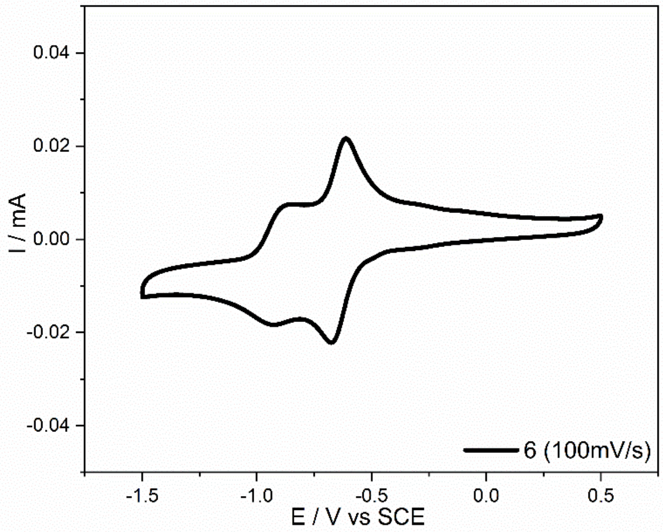

Figure S2. Cyclic voltammetry 100mV s^{-1} with a concentration of 0.1M of the monomer **6,** in 0.1 M tetrabutylammonium perchlorate (TBAP) in acetonitrile and saturated N_2 atmosphere.

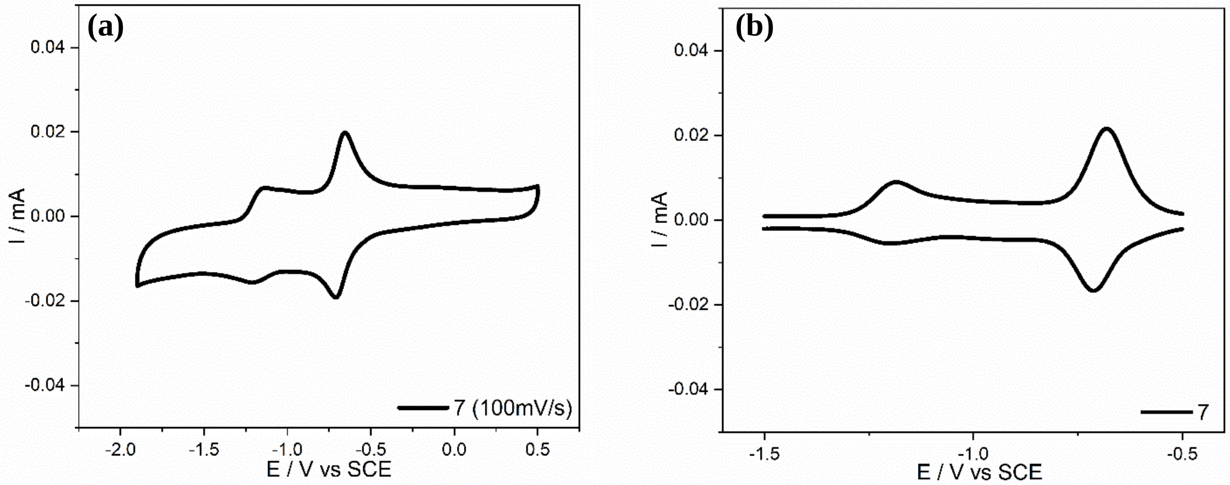

Figure S3. (a) Cyclic voltammetry 100mV s^{-1} and (b) square wave voltammetry (SWV) with a concentration of 0.1M of the monomer **7**, in 0.1 M tetrabutylammonium perchlorate (TBAP) in acetonitrile and saturated N_2 atmosphere.

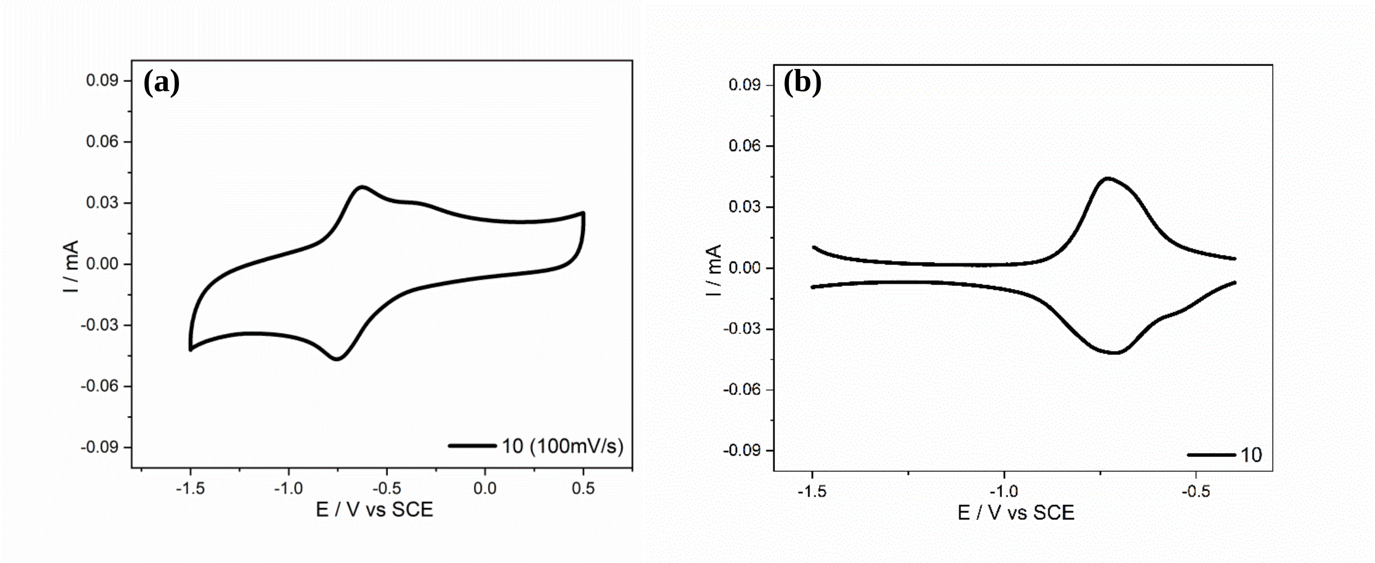

Figure S4. (a) Cyclic voltammetry 100mV s^{-1} and (b) square wave voltammetry (SWV) with a concentration of 0.1M of the heterodimer **10**, in 0.1 M tetrabutylammonium perchlorate (TBAP) in acetonitrile and saturated N_2 atmosphere.

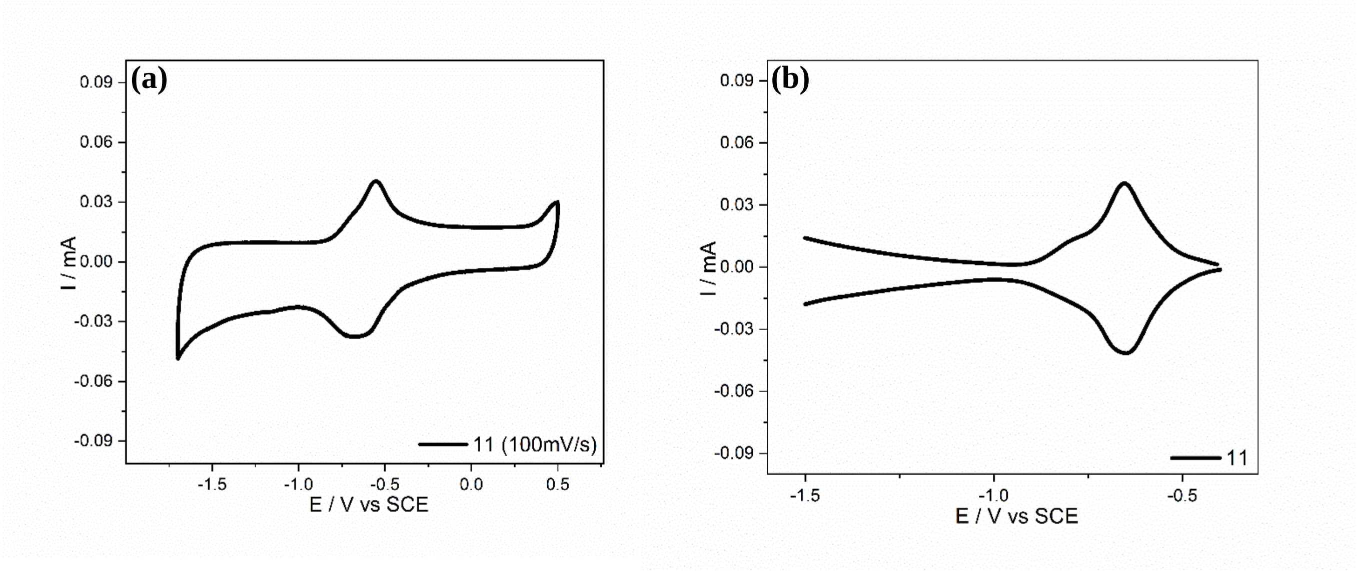

Figure S5. (a) Cyclic voltammetry 100mV s^{-1} and (b) square wave voltammetry (SWV) with a concentration of 0.1M of the heterodimer **11**, in 0.1 M tetrabutylammonium perchlorate (TBAP) in acetonitrile and saturated N_2 atmosphere.

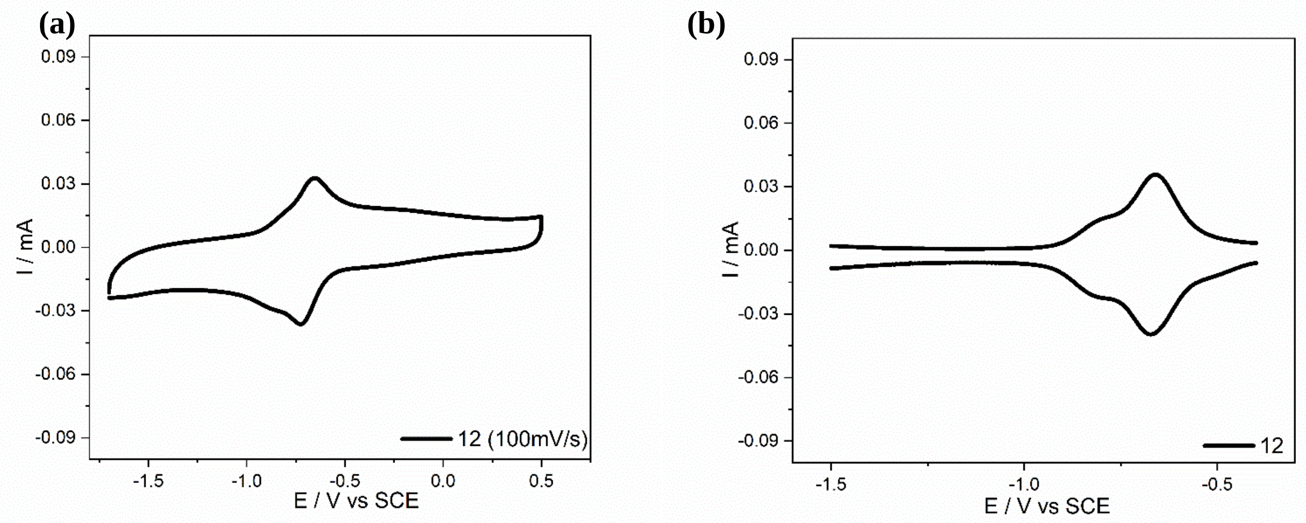

Figure S6. (a) Cyclic voltammetry 100mV s^{-1} and (b) square wave voltammetry (SWV) with a concentration of 0.1M of the heterodimer **12**, in 0.1 M tetrabutylammonium perchlorate (TBAP) in acetonitrile and saturated N_2 atmosphere.

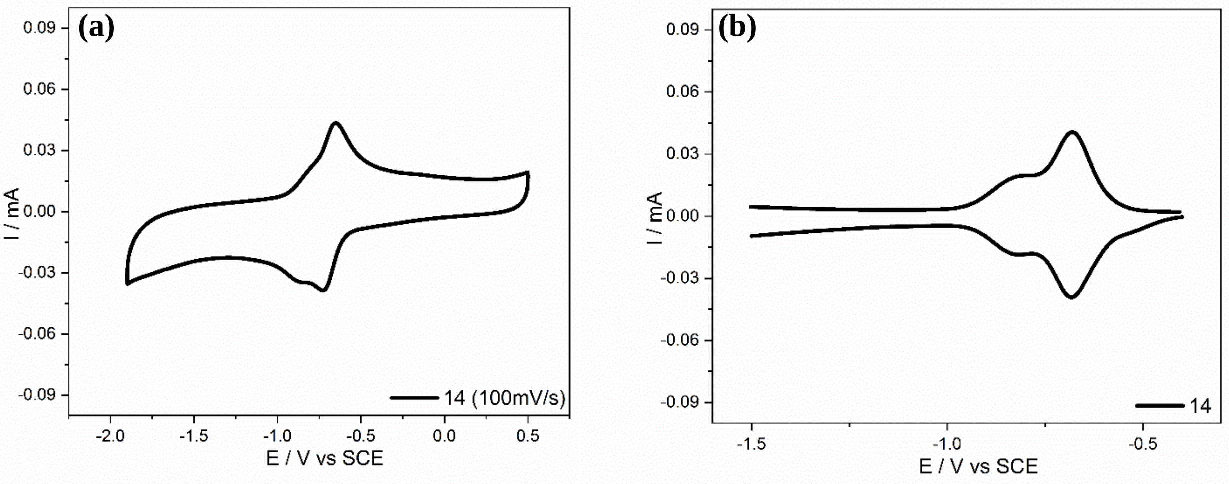

Figure S7. (a) Cyclic voltammetry 100mV s^{-1} and (b) square wave voltammetry (SWV) with a concentration of 0.1M of the homodimer **14**, in 0.1 M tetrabutylammonium perchlorate (TBAP) in acetonitrile and saturated N_2 atmosphere.

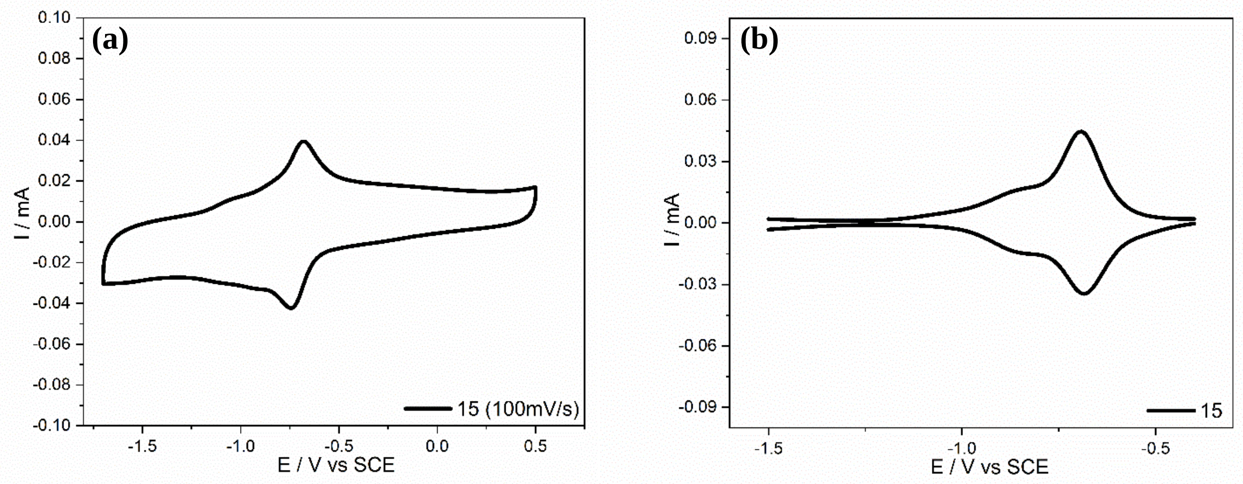

Figure S8. (a) Cyclic voltammetry 100mV s^{-1} and (b) square wave voltammetry (SWV) with a concentration of 0.1M of the homodimer **15**, in 0.1 M tetrabutylammonium perchlorate (TBAP) in acetonitrile and saturated N_2 atmosphere.
